# Surgical practices in emergency umbilical hernia repair and implications for trial design

**DOI:** 10.1007/s10029-024-03165-y

**Published:** 2024-09-21

**Authors:** Josephine Walshaw, Neil J. Smart, Natalie S. Blencowe, Matthew J. Lee

**Affiliations:** 1 Leeds Institute of Medical Research, St James’s University Hospital, University of Leeds, Beckett Street, LS9 7TF Leeds, UK; 2 Leeds Institute of Emergency General Surgery, St James’s University Hospital, Leeds, UK; 3 Department of Colorectal Surgery, Royal Devon and Exeter NHS Foundation Trust, Exeter, UK; 4 Bristol Centre for Surgical Research, Population Health Sciences, University of Bristol, Bristol, UK; 5 Institute for Applied Health Research, College of Medical and Dental Sciences, University of Birmingham, Birmingham, UK; 6 Department of Trauma and Emergency General Surgery, University Hospitals Birmingham NHS Foundation Trust, Birmingham, UK

**Keywords:** Hernia, Umbilical hernia, Emergency

## Abstract

**Introduction:**

There is variation in the investigation, management, and surgical technique of acutely symptomatic umbilical hernias and optimal strategies remain to be established. This survey aimed to identify key variables influencing decision-making and preferred surgical techniques in emergency umbilical hernia care to help inform trial design and understand potential challenges to trial delivery.

**Methods:**

A survey was distributed to surgeons through social media, personal contacts, and ASGBI lists. It comprised five sections: (i) performer of repair, (ii) repair preferences, (iii) important outcomes, (iv) perioperative antibiotic use, and (v) potential future trial design.

**Results:**

There were 105 respondents, of which 49 (46.6%) were consultants. The median largest defect surgeons would attempt to repair with sutures alone was 2 cm (IQR 2–4 cm). In the acute setting, the most common mesh preferences are preperitoneal plane placement (*n* = 61, 58.1%), with synthetic non-absorbable mesh (*n* = 72, 68.6%), in clean (*n* = 41, 39.0%) or clean-contaminated (*n* = 52, 49.5%) wounds. Respondents believed suture repair to be associated with better short-term outcomes, and mesh repair with better long-term outcomes. Pre-/intra-operative antibiotics were very frequently given (*n* = 48, 45.7%) whilst post-operative antibiotics were rarely (*n* = 41, 39%) or very rarely (*n* = 28, 26.7%) given. The trial design felt to most likely influence practice is comparing mesh and suture repair, and post-operative antibiotics versus no post-operative antibiotics. Respondents indicated that to change their practice, the median difference in surgical site infection rate and recurrence rate would both need to be 5%.

**Conclusion:**

This survey provides insight into surgical preferences in emergency umbilical hernia management, offering guidance for the design of future trials.

**Supplementary Information:**

The online version contains supplementary material available at 10.1007/s10029-024-03165-y.

## Introduction

Acutely symptomatic umbilical hernias are a common acute surgical presentation, with around 2,700 requiring emergency surgery in England each year [1, 2]. There is minimal guidance for the management of emergency hernias, with variations existing in the investigation, surgical repair techniques, and perioperative care [3]. This variability is compounded by a paucity of high-quality data across all aspects of emergency umbilical hernia repair, meaning optimal strategies remain to be established. There is a critical need for randomised controlled trials (RCTs) to be undertaken to address key uncertainties such as timing and technical nuances of repair [4].

Prior to conducting an RCT, it is important to understand the nuances of current clinical practice and gauge where clinical equipoise lies. Understanding clinical equipoise is essential as it directly impacts the feasibility and execution of a trial, particularly in surgical settings [5]. A survey of clinicians can facilitate this by elucidating the prevalence and delivery of common interventions or co-interventions, thus defining the variability in current practice. They can also be used to highlight preferences among clinicians and guide sample size calculations by informing minimally important clinical differences. Whilst surveys have previously been conducted in the field, these were designed to understand practice across a range of hernia types [6]. We are not aware of any survey which has been conducted to inform the design of a trial in emergency hernia presentations.

This survey aims to identify key variables that influence surgeon decision-making and preferred surgical techniques in emergency umbilical hernia care. By elucidating these factors, the survey will provide valuable insights to inform the design of future trials and to address potential challenges to trial delivery.

## Methods

### Survey design

An electronic survey was designed to assess surgeons’ preferences regarding various aspects of the treatment of acutely symptomatic umbilical hernias and to help understand potential challenges to the delivery of an RCT. The survey is reported following the Checklist for Reporting Results of Internet E-Surveys (CHERRIES) [7]. Responses were collected in a Google Form. NHS Research Ethics Committee approval is not required for surveys of this nature [8].

### Scope of survey

The survey comprised 21 questions divided into five sections: (i) performer of repair, (ii) repair preferences, (iii) important outcomes, (iv) perioperative antibiotic use, and (v) potential future trial design (Appendix A). Responses were anonymous, and participants were aware that the purpose of the survey was to inform the design of a future RCT.

The questions explored the management of primary umbilical hernias in the emergency setting through multichoice, matrix, and free-text questions. The survey questions were not randomised and there was no adaptive questioning.

#### Performer of repair

This section aimed to identify who is most likely to perform an emergency primary umbilical hernia repair, in terms of level of surgical training.

#### Repair preferences

The repair preferences section was designed to help identify potential inclusion and exclusion criteria. This explored mesh use (including placement, type, and wound contamination), and suture use (including suture material and largest defect they would attempt to close in the emergency setting). For clarity of questioning, definitions and/or images were used for mesh placement (question 4) and Centres for Disease Control and Prevention (CDC) wound classification (question 5) [9].

#### Important outcomes

The important outcomes section was designed to help identify a primary outcome that is relevant to both patients and the population and also to inform statistical design. Participants were asked to select the three most important outcomes in order of preference and also whether they thought suture or mesh repair would confer more favourable outcomes.

#### Perioperative antibiotic use

Surgeons’ preference for pre/intra-operative and post-operative antibiotics was ascertained using a 5-point Likert Scale (‘very rarely’, ‘rarely’, ‘occasionally’, ‘frequently’, ‘very frequently’). Factors influencing antibiotic use and duration were explored through free-text answers.

#### Potential future trial design

**Fig. 1:**
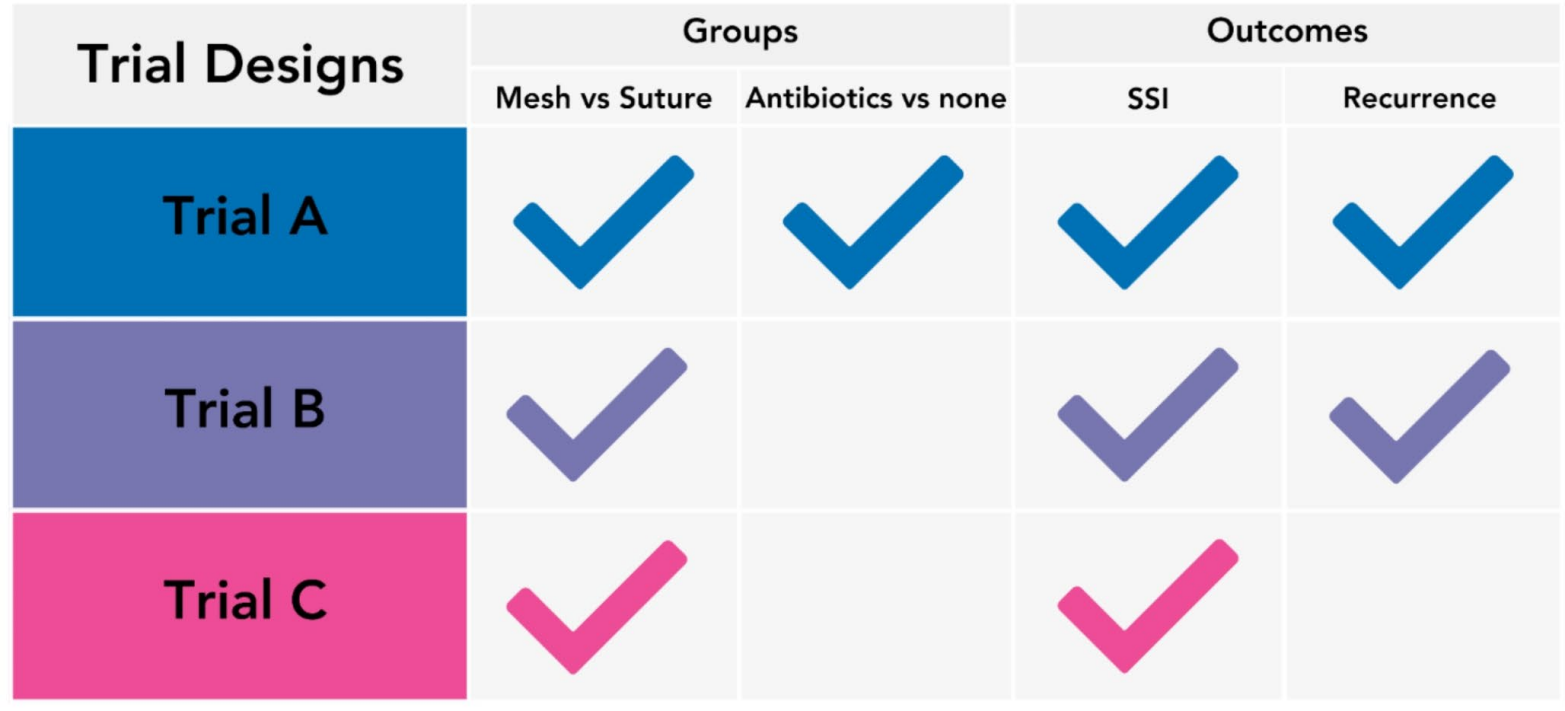
Proposed trial designs for emergency umbilical hernia repair. SSI = Surgical Site Infection

The study team proposed three trial designs for emergency umbilical hernia repair to be undertaken in adult patients aged ≥ 18 years presenting acutely with incarcerated, strangulated, or obstructed primary umbilical or ventral hernias (Fig. 1). Two hypothetical randomisations were proposed: (i) Mesh versus suture repair (with type and position of mesh at the surgeons’ discretion), and (ii) post-operative antibiotics versus no post-operative antibiotics. Questions explored which trial design surgeons felt would most influence clinical practice, willingness to recruit, and the percentage difference required in the outcomes to influence practice.

### Pilot testing

The functionality and ease of use of the electronic survey were tested by the members of the study team before distribution.

### Distribution

The survey was distributed to surgeons through social media, personal contacts, and the Association of Surgeons of Great Britain and Ireland (ASGBI) mailing lists. A brief introduction explained the purpose of the survey and its length. Participation was confidential and voluntary, and completion was interpreted as consent. The survey was open for six weeks between 1st November 2023 and 15th Dec 2023.

### Participants

Responses were eligible if the participant was a surgeon at core surgical training level (or equivalent) or above.

### Analysis

Only completed questionnaires were included in the analysis. Descriptive statistics (using IBM SPSS Statistics (Version 29.0.1.0) [10]) were used to summarise quantitative outcomes including frequencies and percentages for categorical data, and median and interquartile ranges for continuous data. No subgroup analyses were performed. Free-text responses were reviewed by the study team following the principles of thematic analysis.

## Results

### Summary

A total of 105 total survey responses were received, with 49 participants (46.6%) identified as consultant surgeons, 52 (49.5%) as registrars or equivalent, and 4 (3.8%) as core surgical trainees or equivalent.

### Performer of repair

The majority of respondents (*n* = 84/105, 80.0%) indicated that a registrar or equivalent would most likely perform the emergency repair of a primary umbilical hernia in their hospital, while 20 respondents (19.0%) stated a consultant surgeon would perform the repair.

### Repair preferences

**Fig. 2:**
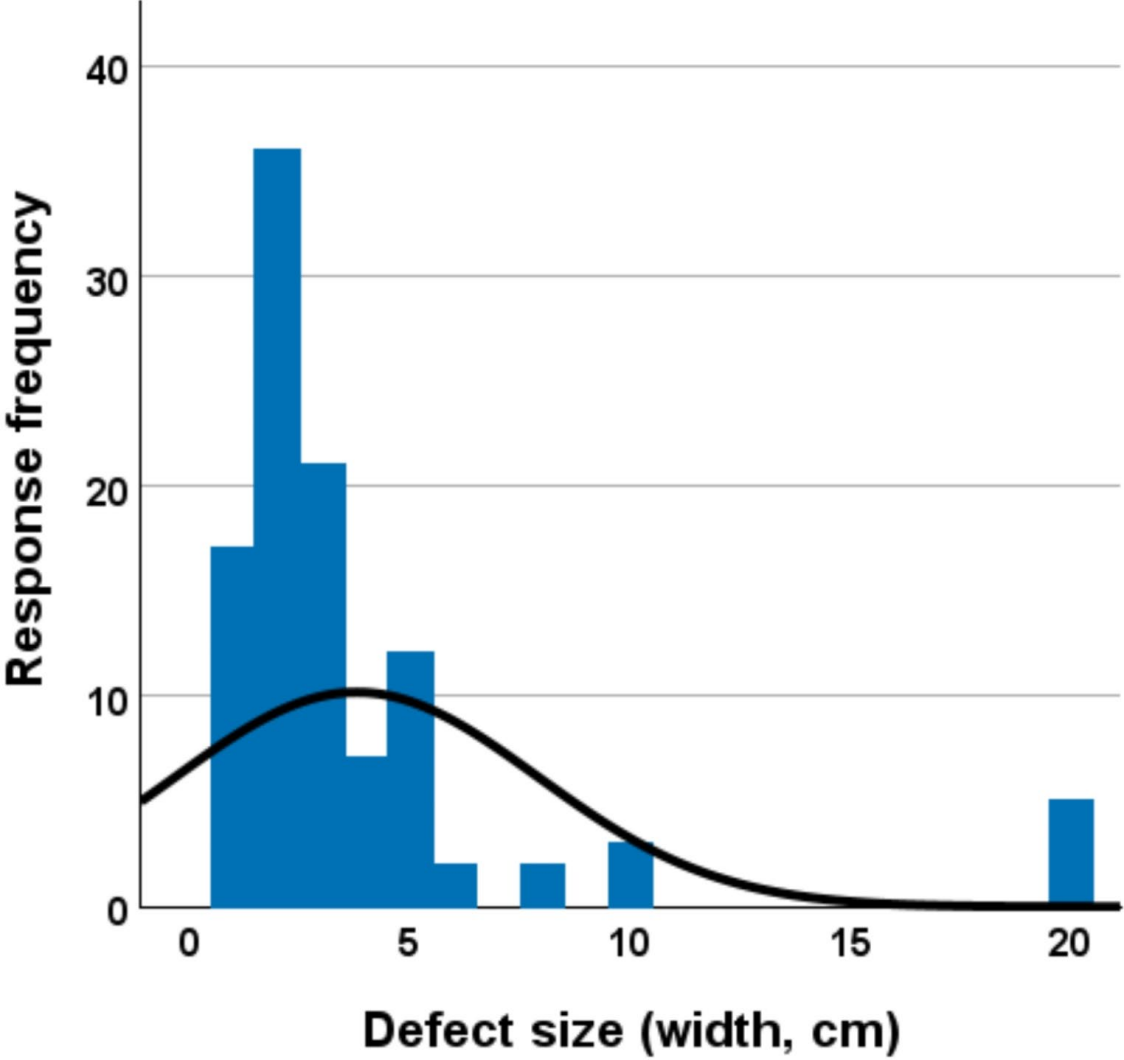
Response distribution for the largest defect (width, cm) surgeons would attempt to repair with sutures alone in the emergency setting

The median largest defect (width) surgeons would attempt to repair with sutures alone in the emergency setting was 2 cm (IQR 2–4 cm) (Fig. 2). The most common mesh preferences were preperitoneal plane placement (*n* = 61/105, 58.1%), with synthetic non-absorbable mesh (*n* = 72/105, 68.6%), in clean (*n* = 41/105, 39.0%) or clean-contaminated (*n* = 52/105, 49.5%) wounds. If a suture repair was to be performed, Prolene (*n* = 43/105, 41%) or Ethilon/Nylon (*n* = 39/105, 37.1%) were the preferred suture materials (Table 1).

**Table 1:** Repair preferences for emergency repair of a primary umbilical hernia

Questions	Responses (*n*, %)	
If you do use a mesh in the acute setting, in which plane do you prefer to place it?	Onlay	9 (8.6%)
	Retrorectus	22 (21.0%)
	Pre-peritoneal	61 (58.0%)
	Intra-peritoneal	8 (7.6%)
	No preference	5 (4.8%)
What is the greatest level of contamination where you would still consider placing a mesh?	CDC 1 (clean)	41 (39.0%)
	CDC 2 (clean-contaminated)	52 (49.5%)
	CDC 3 (contaminated)	11 (10.5%)
	CDC 4 (dirty/infected)	1 (1.0%)
What is your preferred type of mesh for emergency repair of primary umbilical hernia?	Synthetic non-absorbable (e.g. prolene)	72 (68.6%)
	Synthetic absorbable (e.g. vicryl)	11 (10.5%)
	Composite	17 (16.2%)
	Biologic	0 (0.0%)
	Unsure	5 (4.8%)
What is your preferred suture material for emergency repair of primary umbilical hernia? (i.e. for suture only repair)	Prolene	43 (41.0%)
	PDS	15 (14.3%)
	Ethilon/Nylon	39 (37.1%)
	Ethibond	8 (7.6%)

### Important outcomes

**Fig. 3:**
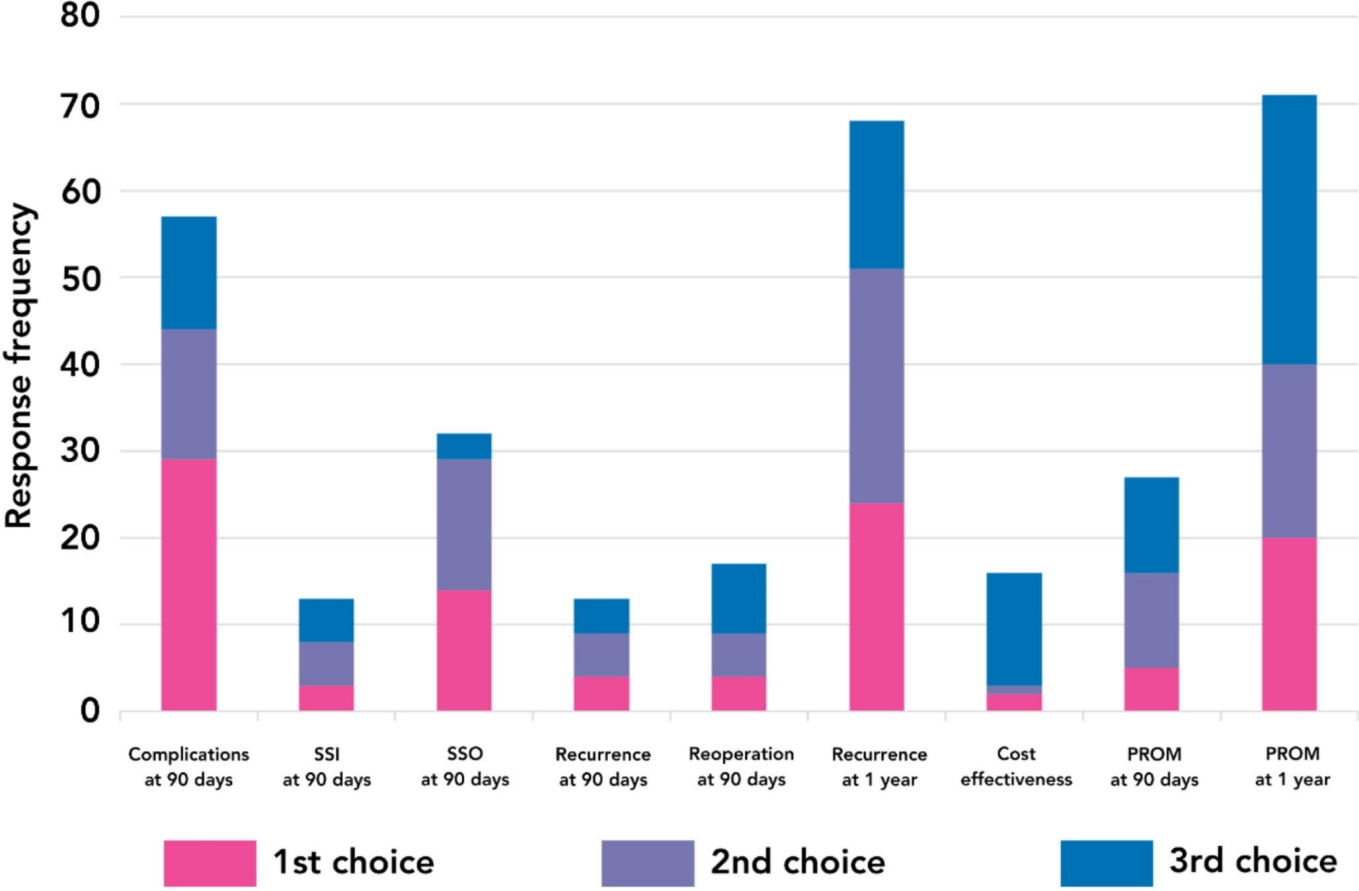
Responses frequency for the first, second, and third choice of ‘most important outcome’ for a trial of umbilical hernia repair in the emergency setting. SSI = Surgical Site Infection. SSO = Surgical Site Occurrence. PROM = Patient Reported Outcome Measure

**Fig. 4:**
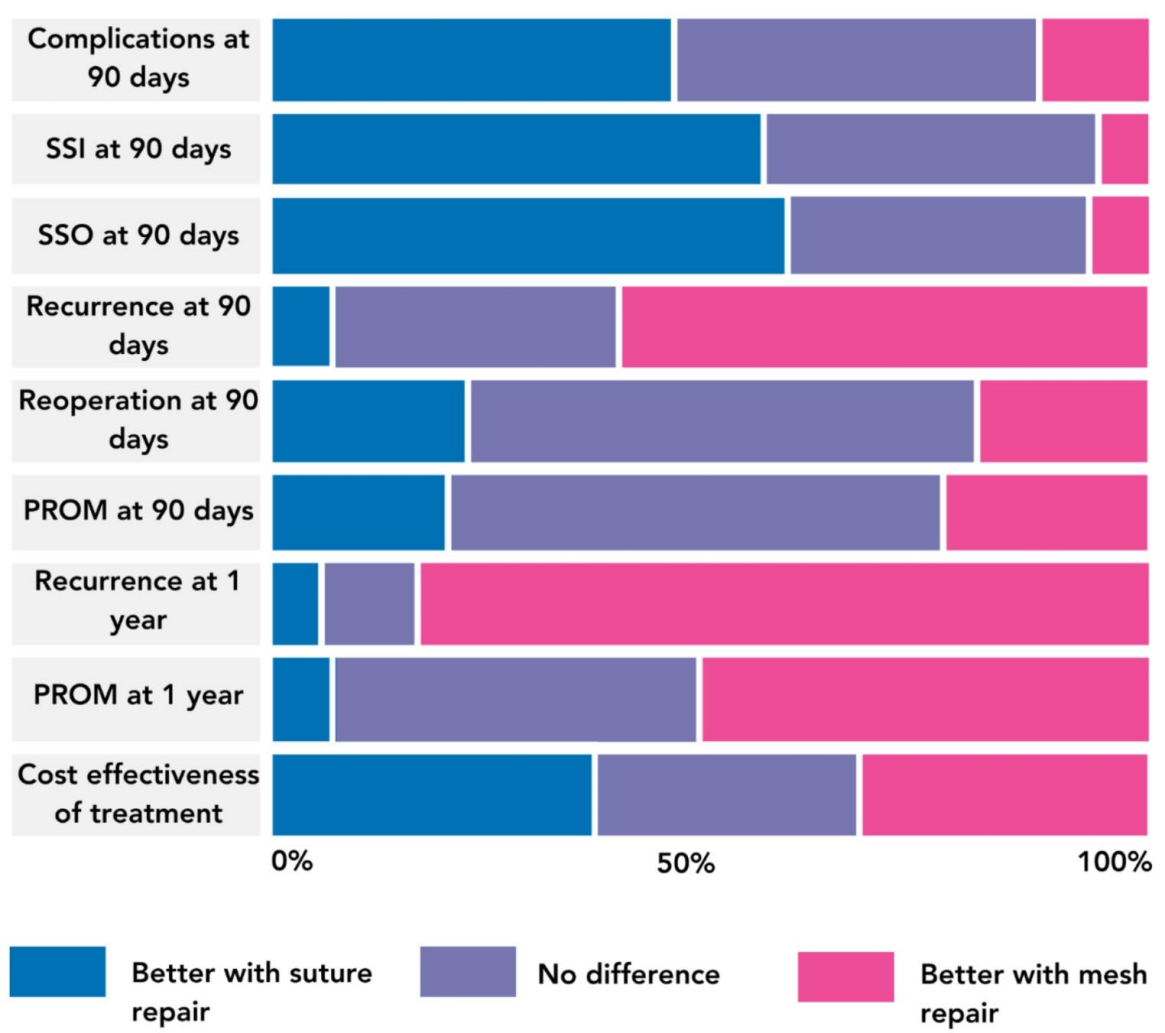
Perspectives on clinical outcomes associated with suture or mesh-based umbilical hernia repair in the emergency setting. SSI = Surgical Site Infection. SSO = Surgical Site Occurrence. PROM = Patient Reported Outcome Measure

Surgeons ranked complications at 90 days, recurrence at 1 year, and patient-reported outcome measures (PROM) at 1 year as the highest priority outcomes for a future study (Fig. 3). Respondents believed suture repair to be associated with better short-term outcomes, including complications at 90 days (*n* = 46/105, 43.8%), SSI at 90 days (*n* = 59/105, 56.2%), and surgical site occurrence (SSO) at 90 days (*n* = 62/105, 59.0%). Mesh repair was felt to be associated with better long-term outcomes including recurrence at 1 year (*n* = 89/105, 84.8%) and patient-reported outcomes at 1 year (*n* = 55/105, 52.4%) (Fig. 4).

### Perioperative antibiotic use

Pre- and intra-operative antibiotics were very frequently (*n* = 48/105, 45.7%) or frequently (*n* = 31/105, 29.5%) given to patients undergoing emergency hernia repairs. Post-operative antibiotics were rarely (*n* = 41/105, 39%) or very rarely (*n* = 28/105, 26.7%) given. Factors influencing decisions to use antibiotics included wound contamination (*n* = 38), hernia content (*n* = 28), and mesh use (*n* = 27).

24 participants (24.5%) stated they would give no post-operative antibiotics. Of those using post-operative antibiotics, 42 participants (42.9%) would give 5 days of antibiotics. The most common preferred empirical antibiotic for contaminated wounds after hernia repair was co-amoxiclav (*n* = 67/105, 63.8%).

### Potential future trial design

The trial design felt to most likely influence practice was a factorial design comparing mesh and suture repair and also antibiotics versus no antibiotics. The preferred co-primary outcome was SSI at 90 days and hernia recurrence at 1 year.

48 (45.7%) of respondents provided free-text comments for important factors in a trial of emergency hernia repair approaches. Analysis of these responses identified five key themes: (i) Patient/hernia factors, (ii) Pre-operative investigations, (iii) Operative factors, (iv) Post-operative outcomes, (v) Trial design (Table 2).

**Table 2:** Identified themes of factors important in a trial of emergency umbilical hernia repair

Themes	Sub-themes	Frequency (number)	Example responses
**Patient/hernia factors** (*n* = 17)	Co-morbidities	8	● Patient factors (weight, smoking, diabetic, previous surgery). ● Previous surgery and ability to place a pre peritoneal mesh would be very influential. ● Size, content (bowel vs. omentum), presence of bowel obstruction, whether hernia is primary or recurrent.
	Hernia factors	9	
**Pre-operative investigations** (*n* = 1)	Imaging	1	● Need to consider for whom preoperative CT would be relevant.
**Operative factors** (*n* = 20)	Repair choice	5	● Needs to be recognised that the circumstances on when to use suture or mesh are different. Of course, clean, >2 cm defect, mesh is best. But if there’s any concern of contamination, almost regardless of the defect size, I opt for suture repair. ● Whether the mesh is fixed (and with what suture) should be included. ● Position and type of mesh could be influential cofounders and wonder whether more standardisation of that would be helpful in the trial design. ● Ability to close defect. ● Drain or no drain? ● Need to encompass laparoscopic/ robotic approaches.
	Mesh factors	10	
	Contamination	5	
	Wound closure	3	
	Drain use	2	
	Minimally invasive approach	4	
**Post-operative outcomes** (*n* = 8)	Patient-reported	1	● Patient-reported outcomes should be more prominent. ● With emergency hernias the outcome that really matters is re-attendance to health services with problems related to their hernia. Recurrence is one thing but if patients aren’t re-presenting with them then it is less of a problem than if patients are re-presenting with post-op pain with no recurrence. ● Longer term (5y) recurrence rate.
	Complications	4	
	Follow up length	3	
**Trial design** (*n* = 10)	Standardisation	2	● Placing a mesh of any type in any position adds a lot of variables, would you be able to achieve adequate power to answer your question regarding recurrence and ssi. It’s a very pragmatic plan which would likely result in good equipoise but will need a large sample. ● Would randomisation be stratified based on defect size? I think there needs to be a limit. I think getting to 3 cm most would want to place a mesh. ● The range of pts is from > 1 cm to > 10 cm. It will be difficult to compare these pts and to get people to randomise at extremes. Also I think there will be a lot of deviation from allocation. ● There are many patients where we have a clear view of what we think is best. There are a few where decisions are difficult. For a trial to work it needs a pragmatic option.
	Randomisation	5	
	Inclusion criteria	1	
	Pragmatism	3	

Respondents indicated that to change their practice, the median absolute percentage difference in SSI rate and recurrence rate would both need to be 5% (*n* = 52, 49.5% and *n* = 51, 48.6% respectively).

## Discussion

This survey investigated the prevailing practices and preferences regarding emergency umbilical hernia repair among surgeons, offering useful information to guide trial design. Specifically, it delineates the likely operators, which presentations are likely to be randomised, and the outcomes required to alter clinical practice. The survey highlighted repair variations in repair technique, perioperative antibiotics, and the importance of key clinical outcomes, emphasising the need for randomised trials in this field. The most favoured trial design, comparing mesh and suture repair and antibiotics versus no antibiotics, holds promise in addressing several key uncertainties surrounding emergency umbilical hernia repair.

There appears to be consensus amongst surgeons on the key outcomes for a trial in this field. Perceptions of short-term benefits associated with suture repair and long-term benefits with mesh repair underscore the importance of assessing both the immediate and delayed postoperative periods. Assessing SSI at 90 days and hernia recurrence at 1 year is pivotal in gauging the effectiveness and safety of mesh and suture repairs. Additionally, the incorporation of PROMs at 1 year ensures a patient-centred approach, providing insights into the functional and quality of life outcomes that hold paramount importance to patients. It is vital that outcomes and equipoise are explored with patients prior to embarking on a definitive trial.

Although surgical site infection (SSI) is frequent in emergency umbilical hernia repair [11, 12], clear guidance is lacking regarding antibiotic prophylaxis in clean (CDC wound class I) and clean-contaminated (CDC wound class II) hernia repair [13]. Furthermore, the association between the choice of repair material (suture versus mesh and choice of mesh) and the occurrence of SSI, as well as the optimal approach to antibiotics prophylaxis remain to be established [11, 14, 15]. The survey findings indicate that post-operative antibiotics are less frequently routinely administered compared to pre- and intra-operative antibiotics, with common indications including wound contamination, hernia content, and the use of mesh. Given the uncertainty surrounding the efficacy and cost-effectiveness of post-operative antibiotics in preventing SSI in emergency umbilical hernia repair, randomising patients to receive post-operative antibiotics versus no antibiotics with a hypothesis of no difference in SSI at 90 days would provide valuable insights into the optimal perioperative management strategy and aim to reduce the burden of SSI in emergency umbilical hernia repair.

Hernia recurrence is an important outcome when evaluating the success of hernia repair. In the elective setting, mesh repair has demonstrated efficacy in reducing the risk of recurrence, with no significant difference in the risk of SSI or chronic pain compared to suture repair [16]. A higher rate of hernia recurrence in suture repair has also been noted in the emergency setting, however evidence is lacking [4]. Many recurrences may not become apparent within the initial 90-day postoperative period [3], underscoring the necessity of a follow-up period of at least one year to capture recurrences and assess the longer-term effectiveness of mesh and suture repair performed in the acute setting.

However, the execution of such a trial is not devoid of complexities. Challenges within randomisation, particularly in cases of extreme defect sizes and contamination, necessitate careful consideration and robust criteria to prevent protocol deviations. It appears that despite some variation, a trial comparing mesh and suture repair and also antibiotics versus no antibiotics could potentially be delivered with eligibility criteria of defect width up to 5 cm, and CDC wound classifications ranging from 1 to 3, thus ensuring the inclusion of a representative patient cohort and maximising its generalisability. Furthermore, the variability in mesh placement and type as highlighted in the survey, and patient factors such as BMI, may introduce additional factors that need to be addressed through standardised protocols.

We acknowledge this survey has some limitations. Surveys have inherent bias. These include responder bias, and results being confined to the scope and questions of the survey. The relatively modest sample size and focus on surgeons practising in the UK may limit the generalisability of the findings to broader international contexts. It is important to recognise that surgical practices and healthcare systems vary across different geographical locations, potentially impacting decision-making and treatment approaches. For technical reasons, we are also unable to assess the response rate from eligible participants due to issues with our preferred ‘click counter’. However, it is worth noting that we received a similar number of responses to comparable surveys which were successfully used to inform studies in this field [3, 6]. Moreover, the reliance on self-reported data introduces the potential for interest-related bias. While we did not collect data on the subspecialty interest of the respondents, which limits the ability to analyse how subspecialty focus may influence practice, surgeons volunteering to participate may hold specific interests or perspectives regarding emergency hernia repair, potentially skewing the reported preferences and practices.

Furthermore, the survey did not address the option for emergency minimally invasive management of umbilical hernias. While open surgery currently represents the predominant repair approach in the acute setting [3], there is growing interest in laparoscopic (and indeed robotic) techniques for emergency hernia repair. However, limited outcome data are available for emergency minimally invasive umbilical hernia repair [3, 4], highlighting a gap in the existing literature that warrants further investigation.

The survey findings carry implications for both researchers and policy-makers in the realm of emergency umbilical hernia repair. The identified variations in surgical techniques and perioperative care highlight the need for well-designed RCTs to inform evidence-based practice. Moreover, involving patients and the public in the design and execution of trials is imperative to ensure that research outcomes align with patient preferences and priorities. This enhances the relevance and acceptability of interventions by better addressing the diverse needs and perspectives of patients, ultimately leading to more effective and patient-centric care delivery.

## Conclusion

This survey offers valuable insights into surgical preferences in emergency umbilical hernia management. These findings provide important guidance for the design of future clinical trials in this field, aiming to generate high quality evidence to inform and improve patient care.

## Electronic supplementary material

Below is the link to the electronic supplementary material.

Supplementary Material 1

Supplementary Material 2
