# Supplementary material for: Surgical practices in emergency umbilical hernia repair and implications for trial design
Source: Hernia. 2024 Sep 21;28(6):2333–41. doi: 10.1007/s10029-024-03165-y (PMC11530507; doi:10.1007/s10029-024-03165-y)
Supplement: Supplementary file 1 — Supplementary Material 1 [file 10029_2024_3165_MOESM1_ESM.pdf]

Supplementary Material 1: Electronic Survey

Performer of repair	
1. What is your current level of clinical practice?	☐ Consultant<br>☐ Registrar or equivalent<br>☐ CST or equivalent
2. In your hospital, who is most likely to perform the operation for an emergency repair of a primary umbilical hernia?	☐ Consultant<br>☐ Registrar or equivalent<br>☐ CST or equivalent
**Repair preferences**	
3. What is the largest umbilical defect you would attempt to close with sutures in the emergency setting?	☐ 1cm ☐ 6cm ☐ 11cm ☐ 16cm<br>☐ 2cm ☐ 7cm ☐ 12cm ☐ 17cm<br>☐ 3cm ☐ 8cm ☐ 13cm ☐ 18cm<br>☐ 4cm ☐ 9cm ☐ 14cm ☐ 19cm<br>☐ 5cm ☐ 10cm ☐ 15cm ☐ 20cm
4. If you do use a mesh in the acute setting, in which plane do you prefer to place it?	☐ I typically prefer ONLAY<br>☐ I typically prefer RETRORECTUS<br>☐ I typically prefer PREPERITONEAL<br>☐ I typically prefer INTRAPERITONEAL<br>☐ No preference
5. What is the greatest level of contamination where you would still consider placing a mesh?	☐ CDC 1 (Clean)<br>☐ CDC 2 (Clean-contaminated)<br>☐ CDC 3 (Contaminated)<br>☐ CDC 4 (Dirty/Infected)
6. What is your preferred type of mesh for emergency repair of primary umbilical hernias?	☐ Synthetic non-absorbable (e.g. prolene)<br>☐ Synthetic absorbable (e.g vicryl)<br>☐ Composite<br>☐ Biologic<br>☐ Unsure
7. What is your preferred suture material for emergency repair of a primary umbilical hernia? (i.e. for suture only repair)	☐ Prolene<br>☐ PDS<br>☐ Ethilon/Nylon<br>☐ Other_________
**Important outcomes**	
8. Which outcome do you think is MOST important?	☐ Complications at 90 days<br>☐ Surgical site infection at 90 days

	☐ Surgical site occurence (infection + seroma, haematoma) at 90 days<br>☐ Recurrence at 90 days<br>☐ Reoperation at 90 days<br>☐ Recurrence at 1 year<br>☐ Cost-effectiveness of treatment<br>☐ Patient-reported outcome measure for hernia at 90 days<br>☐ Patient-reported outcome measure for hernia at 1 year
9. Which outcome is SECOND most important?	☐ Complications at 90 days<br>☐ Surgical site infection at 90 days<br>☐ Surgical site occurence (infection + seroma, haematoma) at 90 days<br>☐ Recurrence at 90 days<br>☐ Reoperation at 90 days<br>☐ Recurrence at 1 year<br>☐ Cost-effectiveness of treatment<br>☐ Patient-reported outcome measure for hernia at 90 days<br>☐ Patient-reported outcome measure for hernia at 1 year
10. Which outcome is THIRD most important?	☐ Complications at 90 days<br>☐ Surgical site infection at 90 days<br>☐ Surgical site occurence (infection + seroma, haematoma) at 90 days<br>☐ Recurrence at 90 days<br>☐ Reoperation at 90 days<br>☐ Recurrence at 1 year<br>☐ Cost-effectiveness of treatment<br>☐ Patient-reported outcome measure for hernia at 90 days<br>☐ Patient-reported outcome measure for hernia at 1 year

11. Please indicate whether you feel these outcomes are more likely to be better with mesh or suture based options.

	Better with suture	No difference	Better with mesh	Unsure
Complications at 90 days	☐	☐	☐	☐
Surgical site infection at 90 days	☐	☐	☐	☐
Surgical site occurrence at 90 days	☐	☐	☐	☐
Recurrence at 90 days	☐	☐	☐	☐
Reoperation at 90 days	☐	☐	☐	☐
Recurrence at 1 year	☐	☐	☐	☐
Cost effectiveness of	☐	☐	☐	☐

	treatment				
	Patient reported outcomes at 90 days	▢	▢	▢	▢
	Patient reported outcomes at 1 year	▢	▢	▢	▢

**Perioperative antibiotic use**	
12. How often do you give pre/intra-operative antibiotics before emergency hernia repair?	▢ Very rarely<br>▢ Rarely<br>▢ Occasionally<br>▢ Frequently<br>▢ Very frequently
13. How often do you give post-operative antibiotics to patients after emergency hernia repair?	▢ Very rarely<br>▢ Rarely<br>▢ Occasionally<br>▢ Frequently<br>▢ Very frequently
14. When would you consider using pre/intra-operative or post-operative antibiotics?	____
15. How long a course of antibiotics do you give post-operatively? (in days)	____
16. What are your preferred empirical antibiotics for contaminated wounds after hernia repair?	▢ Cefuroxime and metronidazole<br>▢ Co-amoxiclav<br>▢ Flucloxacillin<br>▢ Tazocin<br>▢ Other________________
**Trial design**	
17. Which trial design would be most likely to influence your practice?	▢ **Trial A** - Randomised to open mesh vs suture repair AND also to post-operative antibiotics vs no antibiotics. Test hypothesis that there is no difference in SSI rates at 90 days AND mesh repair provides lower rates of recurrence at 1 year.<br><br>▢ **Trial B** - Randomised to open mesh vs suture repair. Test hypothesis that there is no difference in SSI rates at 90 days AND mesh repair provides lower rates of recurrence at 1 year.<br><br>▢ **Trial C** - Randomised to open mesh vs suture repair. Test hypothesis that there is no difference in SSI rates at 90 days.

18. Which trial would you be willing to recruit to?	<table><tr><th></th><th>Would randomise</th><th>Unsure</th><th>Would not randomise</th></tr><tr><td>Trial A</td><td>▢</td><td>▢</td><td>▢</td></tr><tr><td>Trial B</td><td>▢</td><td>▢</td><td>▢</td></tr><tr><td>Trial C</td><td>▢</td><td>▢</td><td>▢</td></tr></table>
19. Do you have any other comments about what is important in a trial of repair approaches in emergency primary umbilical hernia repair?	____
20. What percentage difference in surgical site infection would a trial have to show for you to change your practice?	____
21. What percentage difference in hernia recurrence would a trial have to show for you to change your practice?	____
